# Thermal Treatment Prevents Effects of Downward Loads on the Screw-In Force Generation and Canal-Centering Ability of Nickel–Titanium Rotary Instruments

**DOI:** 10.3390/ma18153610

**Published:** 2025-07-31

**Authors:** Keiichiro Maki, Arata Ebihara, Yanshan Luo, Yuka Kasuga, Hayate Unno, Satoshi Omori, Shunsuke Kimura, Takashi Okiji

**Affiliations:** 1 Department of Pulp Biology and Endodontics, Division of Oral Health Sciences, Graduate School of Medical and Dental Sciences, Institute of Science Tokyo, 1-5-45 Yushima, Bunkyo-ku, Tokyo 113-8549, Japan; a.ebihara.endo@tmd.ac.jp (A.E.); luo.yanshan@tmd.ac.jp (Y.L.); y.kasuga.endo@tmd.ac.jp (Y.K.); h.unno.endo@tmd.ac.jp (H.U.); s.omori.endo@tmd.ac.jp (S.O.); s.kimura.endo@tmd.ac.jp (S.K.); t.okiji.endo@tmd.ac.jp (T.O.); 2 Department of Endodontics, The Nippon Dental University School of Life Dentistry at Tokyo, 1-9-20 Fujimi, Chiyoda-ku, Tokyo 102-8159, Japan

**Keywords:** nickel–titanium rotary instrument, root canal treatment, canal-centering ratio, torque, vertical force, downward load, instrumentation time, thermal treatment

## Abstract

This study aimed to examine how downward load applied during instrumentation affects the stress generation and shaping properties in thermally treated and non-treated NiTi rotary instruments. ProTaper Universal (PTU; non-thermally treated) and ProTaper Gold (PTG; thermally treated) were used to prepare J-shaped canals in resin blocks. Load-controlled automated instrumentation and torque/force sensing devices were employed with preset downward loads of 1, 2, or 3 N (n = 10 each). The torque/force, instrumentation time, and canal-centering ratio were measured and analyzed using two-way or one-way analysis of variance with Tukey’s test (α = 0.05). In the PTU-1N group, instrumentation was not completed because a ledge was formed in all canals. The PTU-3N group showed significantly greater upward force (screw-in force) and clockwise torque, along with a significantly smaller canal-centering ratio (less deviation) at the apical 0 mm level, than the PTU-2N group (*p* < 0.05). The downward load did not influence the instrumentation time (*p* > 0.05). In the PTG groups, these effects of downward load on the force generation and canal-centering ratio were not significant (*p* > 0.05). In the non-thermally treated PTU instruments, greater downward loads enhanced screw-in force while decreasing apical canal deviation; however, these effects were abolished in the thermally treated PTG instruments. This study highlights the importance of adapting the instrumentation technique with instrument characteristics: thermally treated flexible instruments facilitate smoother use, while stiffer, non-thermally treated ones may require precise control of downward loads.

## 1. Introduction

Root canal treatment is a definitive procedure for managing apical periodontitis, an inflammatory condition of the apical periodontal region resulting from persistent microbial stimuli, which originates from the infected root canal system [1,2]. Successful root canal treatment relies on proper instrumentation in the canal because this process is crucial for mechanically removing infectious substances from the canal, enabling effective irrigation and facilitating the hermetic obturation of the cleaned root canal system [3,4].

Nickel–titanium (NiTi) rotary instruments are increasingly favored for achieving precise and effective root canal instrumentation owing to their superior flexibility [5,6], which enhances their ability to prepare curved canals with less deviation [7,8], as well as their high cutting efficiency [7,9] compared with traditional stainless steel hand instruments. Nevertheless, unforeseen separation within the canal is still a significant issue when using NiTi rotary instruments [10,11]. Therefore, considering that improper manipulation can be a significant cause of instrument fractures [12,13], careful manipulation of NiTi instruments by the operator is essential to fully benefit from the advantages of these instruments and ensure the desired outcomes [11]. Proper manipulation of NiTi rotary instruments, including appropriate application of apical pressure and controlled up-and-down motions, is required to provide safe and high-quality care [11,12,13,14,15,16,17]. However, there is limited information on how these operator-dependent factors affect instrumentation outcomes, largely because evaluating their impact under standardized experimental conditions presents significant challenges. Using automated instrumentation, we previously demonstrated that a higher up-and-down speed (pecking speed) achieves better canal-centering ability but generates larger torque/force than a lower pecking speed [12]. Furthermore, a larger pecking amplitude (the amplitude of the up-and-down motion) is reported to increase the screw-in force [14,15] and improve the canal-centering ability [15]. However, its impact on cyclic fatigue resistance varies depending on the specific NiTi instrument employed [15,16,17]. The addition of a brushing motion to the in-and-out motion has been reported to have no impact on the cyclic fatigue resistance [18] and shaping ability [19] of NiTi instruments, and it does not lead to dentin crack formation [20] during instrumentation.

The downward load (insertion pressure) is another crucial operator-dependent factor that must be properly controlled, given that an excessive downward load may cause the instrument to bind tightly to the canal wall, increasing torsional stress and the risk of fracture [13]. We used load-controlled automated instrumentation in another study [13], showing that elevating the downward load during NiTi rotary instrumentation augments the upward force (screw-in force), which can cause sudden threading of the instrument and fracture [21,22]. However, increasing the downward load improves the canal-centering ability and reduces instrumentation time [13].

Significant efforts to improve the fracture resistance of NiTi instruments have led to the development of various new technologies such as thermally treated NiTi alloys [5,6,23] and the adoption of reciprocating motion [24,25,26]. In particular, the thermal treatment alters the phase composition of the NiTi wire, shifting it from an austenite-rich phase to softer and more ductile R- and martensite-rich phases [5,6], thereby enhancing its mechanical flexibility [27,28] and resistance to cyclic fatigue [6,29].

Despite the clinical significance, it remains unclear whether and how operator-dependent factors affect the performance of thermally treated and non-treated NiTi rotary instruments. Therefore, this study aimed to address this gap by examining how applied downward load influences torque/force generation, canal-centering ability, and instrumentation time for both instrument types. The null hypothesis was that the downward load has no impact on the parameters under investigation.

## 2. Materials and Methods

### 2.1. Sample Size Estimation

Based on the data obtained from preliminary experiments and a previous study [13], the required sample size of 10 per group was determined using G*Power software (version 3.1.9.2, Heinrich Heine University, Düsseldorf, Germany), with an effect size of 1.4, α error of 0.05, and power of 0.80.

### 2.2. Automated Root Canal Instrumentation Device

Load-controlled automated root canal instrumentation, rather than operator-controlled instrumentation, and torque/force sensing devices were employed to maintain reproducibility, as described in our previous study [13]. The instrumentation device comprised a torque-controlled, low-speed motor (J Morita, Kyoto, Japan) paired with a motorized testing stand (MX2-500N; Imada, Toyohashi, Japan) [13]. A specially designed handpiece holder was mounted to the stand using an electromagnet, with the holder suspended and balanced by weights on pulleys. When the electromagnet was activated, the handpiece and stage moved together at a rate of 50 mm/min, and when deactivated, the handpiece was released to fall with a downward load of either 1, 2, or 3 N, controlled using the weights.

**Figure 1:**
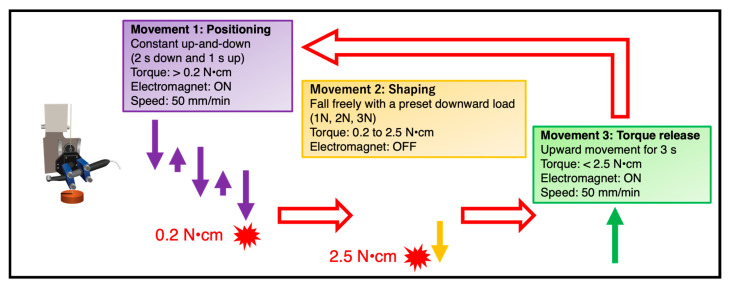
Movements of the root canal instrumentation device.

The handpiece was set to execute one of three movements based on the level of clockwise torque that was sensed by the motor, as previously described (Figure 1) [13]:

Movement 1. When torque was >0.2 N·cm, the electromagnet was activated, causing the handpiece and stage to move downward for 2 s and then upward for 1 s at 50 mm/min. This was the initial movement used to begin instrumentation.

Movement 2. When the torque ranged from 0.2 to 2.5 N·cm, the electromagnet was deactivated, allowing the handpiece to descend freely under the preset downward load of 1, 2, or 3 N.

Movement 3. When the torque exceeded 2.5 N·cm, the electromagnet was activated, and the handpiece moved upward for 3 s at a rate of 50 mm/min. Subsequently, the handpiece performed one of three possible movements based on the torque detected by the motor.

The measuring system contained strain gauges (KFG-2-120-D31-11, Kyowa Electronic Instruments, Tokyo, Japan) for torque measurements and a load cell (LUX-B-ID, Kyowa Electronic Instruments) for vertical force measurements [13]. A resin block with a J-shaped artificial canal (size #15, 0.02 taper, 17 mm length, 45° curvature, Endo Training Bloc, Dentsply Sirona, Ballaigues, Switzerland) was secured to a metal stage connected to the torque/force measurement system. Signal amplification was performed with an amplifier (PCD-400A, Kyowa Electronic Instruments), and the data were processed with data acquisition software (DCS-100A ver.04.91, Kyowa Electronic Instruments).

### 2.3. Root Canal Instrumentation

Instruments with identical geometry, namely ProTaper Universal (PTU; non-thermally treated, Dentsply Sirona) and ProTaper Gold (PTG; made of thermally treated Gold wire, Dentsply Sirona), were chosen as test instruments to eliminate geometry-dependent variables. The resin blocks (n = 60) were flared coronally using the PTG SX instrument (Dentsply Sirona) to a depth of 5 mm from the apex. Following patency verification with the #10 stainless steel hand K-file, the resin blocks were instrumented using PTG S1 (size 18/0.02 taper at the tip) and S2 (size 20/0.04 taper at the tip). Then, the samples were randomly distributed into the PTU and PTG groups, which were further subdivided into the 1 N, 2 N, and 3 N groups, corresponding to downward loads of 1, 2, and 3, respectively (n = 10 per subgroup).

Each canal was instrumented with either PTG or PTU using the automated instrumentation device described above. The F1 instrument (size 20/0.07 taper at the tip), F2 instrument (size 25/0.08 taper at the tip), and F3 instrument (size 30/0.09 taper at the tip) were used in sequence for both groups. The instrumentation process consisted of two steps, starting with instrumentation up to 1 mm short of the working length followed by instrumentation to the full working length. A lubricant (RC Prep, Premier, Plymouth Meeting, PA, USA) was applied throughout the instrumentation process. After each instrument use, the canal was irrigated with 1 mL of distilled water, and the patency was verified using a #10 stainless steel K-file. Each instrument was used in a single canal. Upward and downward forces, along with clockwise torque, were recorded during the instrumentation process, and the maximum values produced by each instrument were identified.

### 2.4. Assessment of the Instrumentation Time and Canal-Centering Ratio

The instrumentation time was defined as the duration between the moment the torque initially surpassed 0.2 N·cm and the completion of the instrumentation process, as previously described [13]. This time was computed using the raw torque data obtained from the torque/force measuring unit.

Canal-centering ratios were evaluated with image analysis software (Photoshop 7.0, Adobe Systems, San Jose, CA, USA), as previously described [12,13]. In brief, digital images taken before and after instrumentation were superimposed, and the resin removed from both the outer and inner walls of the canal was measured at five specific points: 0, 0.5, 1, 2, and 3 mm from the apex. The centering ratio was calculated using the formula (X − Y)/Z, where X represents the amount removed from the outer wall, Y the amount removed from the inner wall, and Z the post-instrumentation canal diameter.

### 2.5. Statistical Analysis

All statistical analyses were conducted using SPSS software (version 27.0, IBM, Armonk, NY, USA). Data normality was confirmed using the Shapiro–Wilk test, and variance homogeneity was verified using Levene’s test. A two-way analysis of variance (ANOVA), followed by Tukey’s post hoc test, was applied to examine the canal-centering ratio, force, torque, and instrumentation time for each instrument. A one-way ANOVA, along with Tukey’s test, was employed to evaluate the total instrumentation time. Statistical significance was set at *p* < 0.05.

## 3. Results

In the PTU-1N group, instrumentation was not completed using F2 or F3 because of the ledge formation that occurred in 10 out of 10 samples. Accordingly, the data from the group PTU-1N were excluded from further analysis. In the other groups, instrumentation was completed without any ledge formation or instrument fractures.

**Figure 2:**
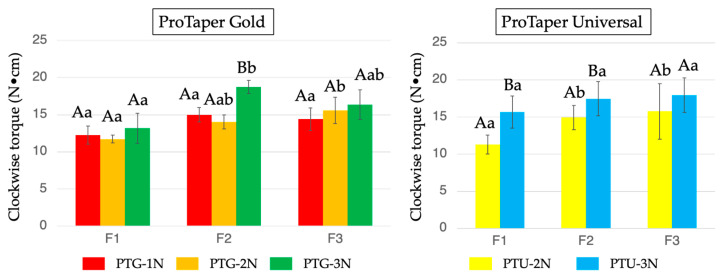
Maximum upward and downward vertical force values detected during PTG and PTU instrumentation using three different instrument sizes (F1, then F2, followed by F3) and three different downward loads (1, 2, and 3 N). Data are shown as the mean and standard deviation (n = 10 for each subgroup using each instrument). Values with different capital letters for the same instrument and values with different small letters for the same downward load are significantly different (*p* < 0.05).

Figure 2 presents the mean maximum vertical force values obtained for PTG and PTU under the three different downward loads. In both groups, a greater downward load led to greater downward vertical force, except for PTU-F3 (*p* < 0.05). Regarding the upward vertical force, representing the screw-in force, no significant difference was observed among all the PTG subgroups or instrument sizes. In contrast, PTU-3N exhibited a significantly larger upward vertical force than PTU-2N (*p* < 0.05).

**Figure 3:**
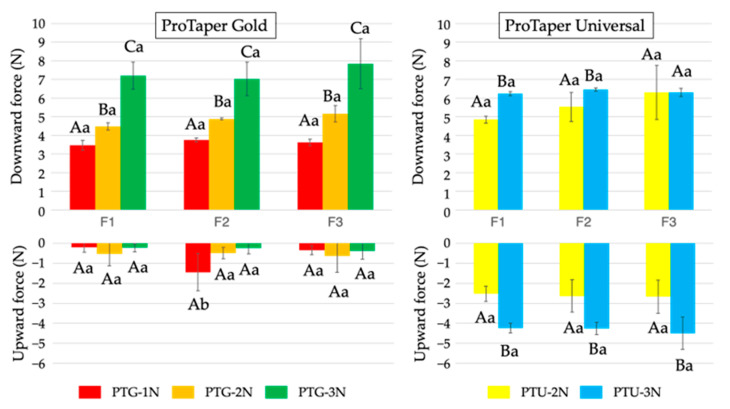
Maximum torque values detected during PTG and PTU instrumentation using three different instrument sizes (F1, then F2, followed by F3) and three different downward loads (1, 2, and 3 N). Data are shown as the mean and standard deviation (n = 10 for each subgroup using each instrument). Values with different capital letters for the same instrument and values with different small letters for the same downward load are significantly different (*p* < 0.05).

With respect to clockwise torque (Figure 3), PTG-3N exhibited a significantly larger torque with F2 than with F1 and F3 (*p* < 0.05), and no significant difference was found between F1 and F3. In the PTU group, the torque for PTU-3N was significantly larger than that for PTU-2N with sizes F1 and F2 (*p* < 0.05).

**Figure 4:**
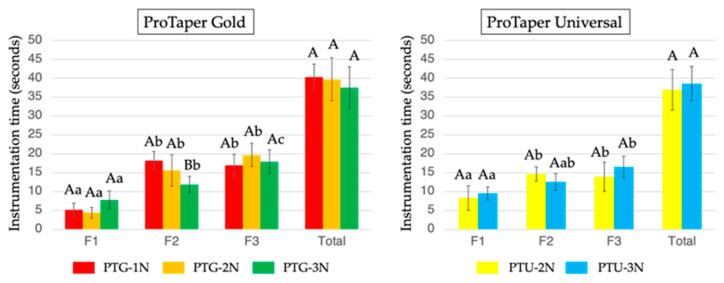
Time required for root canal instrumentation using PTG and PTU with three different instrument sizes (F1, then F2, followed by F3) and three different downward loads (1, 2, and 3 N). Data are shown as the mean and standard deviation (n = 10 for each subgroup using each instrument). Values with different capital letters for the same instrument and values with different small letters for the same downward load are significantly different (*p* < 0.05).

Figure 4 indicates that total instrumentation time did not differ significantly among subgroups for either the PTG or PTU instruments. Within the PTG group, PTG-3N exhibited a significantly shorter instrumentation time with F2 (*p* < 0.05). In the PTU group, no significant differences were found between the subgroups or the instrument sizes.

**Figure 5:**
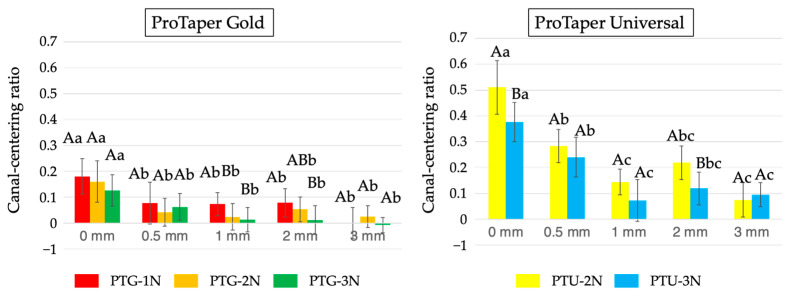
Canal-centering ratios obtained using PTG and PTU with three different instrument sizes (F1, then F2, followed by F3) and three different downward loads (1, 2, and 3 N). Data are shown as the mean and standard deviation (n = 10 for each subgroup using each instrument). Values with different capital letters for the same distance and values with different small letters for the same downward load are significantly different (*p* < 0.05).

Figure 5 displays the canal-centering ratios obtained for PTG and PTU under the different downward loads. In the PTG group, no significant differences were found among the subgroups at 0 and 0.5 mm, but PTG-2N and PTG-3N showed significantly lower canal-centering ratios (less deviation) than PTG-1N at the 1–3 mm levels (*p* < 0.05). In the PTU group, the canal-centering ratio at 0 mm was significantly higher for PTU-2N than for PTU-3N (*p* < 0.05).

## 4. Discussion

The amount of downward load exerted on NiTi rotary instruments is a crucial operator-dependent variable that should be carefully managed to ensure optimal instrumentation outcomes in root canal treatments. Excessive downward load can overload the instruments, leading to torsional stress generation and instrument fracture [11,13]. Additionally, prolonged application of a downward load can lead to fatigue failure because it can promote stress accumulation within the instrument [30]. This study highlights the impact of thermal treatment of NiTi instruments, using PTG (thermally treated) and PTU (non-treated) with the same geometry, on their torque/force generation, canal-centering ability, and instrumentation time under differently downward-loaded instrumentation conditions. The results demonstrated that the downward load had a significant effect on PTU, with larger loads increasing the screw-in force and smaller loads leading to greater canal deviation. Conversely, the impact on PTG was limited. These findings suggest that thermal treatment plays a crucial role in reducing the adverse effects of downward loads on the performance of NiTi rotary instrumentation.

In this study, the downward load-controlled automated root canal instrumentation device, which was first employed in our previous study [13], was used to objectively evaluate how downward load affects instrumentation outcomes. In addition to controlling the downward load, this device can regulate several operator-dependent variables, including the speed and duration of the up-and-down motion [12], and features an automatic retraction mechanism that is activated by the torque generated during instrumentation and detected by the handpiece [13]. These features allowed us to conduct the present study in a standardized and reproducible manner without human intervention and to objectively evaluate the impact of downward load on the outcomes of rotary instrumentation using NiTi instruments with different metallurgical properties.

Two NiTi instruments with identical geometries were employed, but only one was manufactured with heat treatment [28]. Both PTU and PTG have a variable taper over the length of the cutting blade, a convex triangular cross-sectional design, and a noncutting tip [31]. However, PTU is made of conventional NiTi wire (without heat treatment), whereas PTG undergoes a Gold heat treatment process, resulting in significantly improved flexibility [29,31,32]. According to differential scanning calorimetry analysis, PTU has an austenite finish temperature of 21.2 °C, and PTG has an austenite finish temperature of 50.1 °C [28]. This indicates that under clinical conditions (approximately body temperature, 37 °C), PTG exhibits a martensite-rich phase composition. Thus, PTG has higher cyclic fatigue resistance and better canal-centering ability than PTU [33,34].

The downward vertical force and torque affect the safety of NiTi instrumentation because excessive downward vertical force can cause the instrument to bind tightly to the canal wall, leading to torque generation and instrument fracture [11]. For both PTU and PTG instruments, increases in the preset downward load led to corresponding rises in vertical force and torque. This suggests that excessive downward vertical force should be avoided to prevent instrument fracture, regardless of the metallurgical properties of the NiTi rotary instruments.

During NiTi rotary root canal instrumentation, an excessive screw-in force can lead to intracanal instrument fractures [21] because it may cause the instrument to bind to the canal wall suddenly, producing sudden and abrupt torsional stress [21,22]. Studies have demonstrated that the intensity of the screw-in force is influenced by operator-dependent variables such as the speed [12] and depth [14] of pecking motions. The present findings support our previous observation that an increased downward load augments the upward vertical force representing the screw-in force [13]. Considering that the screw-in force is strongly associated with the instrument’s interaction with root dentin [22], it is plausible that a greater downward load results in tighter engagement of the instrument with the canal wall. However, the present study also revealed that the downward load-dependent screw-in force was increased only in the PTU group, suggesting that this is a characteristic of NiTi instruments with lower flexibility. Previous studies [21,22,35] have shown that the flexibility, cross-sectional design, and pitch length influence the magnitude of the screw-in force. PTU and PTG have the same geometric properties but undergo different manufacturing processes, without and with heat treatment, respectively. Thus, the higher flexibility of heat-treated instruments may facilitate a lower screw-in force [22], potentially enabled by a reduction in the internal stress during instrumentation. In other words, NiTi instruments with lower flexibility are more sensitive to the operator’s application of the downward load, emphasizing the need to adopt appropriate operational techniques.

In all groups studied herein, the instrumented root canals exhibited apical transportation toward the outer wall, which is likely attributed to the tendency of NiTi instruments to return to their original straight shape when passing through curved sections of the canal [36,37]. Among the factors related to operation and handling, a faster pecking speed [12] and reciprocating motion (as opposed to continuous rotation) [26] help reduce apical transportation. However, the effect of the downward load applied during instrumentation on the canal-centering ability of NiTi rotary instruments has not been clarified. In this regard, our previous study demonstrated that instrumentation with a smaller downward load results in larger canal-centering ratios in the apical area, indicating a larger degree of apical transportation [13]. The results of the present study are consistent with these findings: PTU-2N showed a higher canal-centering ratio than PTU-3N at the 0 mm apical level (Figure 4). Moreover, all PTU-1N specimens exhibited ledge formation, indicating a loss of the ability to preserve canal curvature under the small-load condition. These results may be attributed to the shorter contact time between the blades and the canal wall in the subgroups subjected to higher downward loads. In contrast, the ledge formation observed in PTU-1N may be due to the lower flexibility of the PTU instrument compared to PTG instruments, which likely caused excessive resin removal from the outer wall as a result of prolonged contact under small-load conditions. The increased load-induced torque caused more frequent activation of the torque-dependent instrument withdrawal mechanism, leading to the release of contact and reduced resin removal from the outer canal wall. It was evident that canal deviation (apical transportation and ledging) was more pronounced for PTU than for PTG, most likely owing to PTG’s greater flexibility [31,38].

In both the PTG and PTU groups, the F2 and F3 instruments tended to require longer instrumentation times, which was assumed to be due to the considerable increase in dimensions from F1 to F2 and from F2 to F3. Instrumentation from S2 (size 20/0.04 taper at the tip) to F1 (size 20/0.07 taper at the tip) involved an increase in taper only. However, from F1 to F2 (size 25/0.08 taper) and from F2 to F3 (size 30 /0.09 taper), both the apical size and taper were increased.

Our previous study using ProTaper NEXT instruments (Dentsply Sirona) [13] demonstrated that a higher downward load led to a shorter total instrumentation time, but no significant differences were observed among instruments of different sizes. In this study, different downward loads had no significant effect on the total instrumentation time, except for PTG with the F2 size. Thus, for both PTU and PTG, a higher downward load has a limited effect on the instrumentation time. In both groups, F2 and F3 showed significantly longer instrumentation times than F1. The amount of resin removal from F1 (#20/0.07 taper) to F2 (#25/0.08 taper) was greater than that from S2 (#20/0.04 taper) to F1 (#20/0.07 taper), which may have influenced the instrumentation time.

In the PTG group, instrumentation was completed in all specimens without any ledge formation, indicating a better ability to preserve the canal curvature than the PTU group. The downward force increased depending on the increase in the downward load, whereas the upward vertical force (screw-in force), torque, and canal-centering ratio were less affected by the magnitude of the downward load. These results suggest that PTG, being more flexible than PTU [29,31,32], is more forgiving to variations in downward load during instrumentation, facilitating easier manipulation and lowering the likelihood of adverse outcomes.

Overall, the present findings indicate that the outcome of instrumentation is more affected by the downward load in non-thermally treated NiTi instruments with lower flexibility than in those with higher flexibility. Thus, NiTi instruments with lower flexibility are less able to adapt to variations in downward load, necessitating thorough training in their clinical use, particularly for operators with limited experience in NiTi rotary instrumentation. If the operator must use NiTi instruments with lower flexibility, the risk may be reduced by using a motor with a reciprocating motion, such as the optimum torque reverse (OTR) motion—a torque-sensitive reciprocal rotation—rather than continuous rotation [26]. OTR motion is reported to produce lower vertical force and torque while maintaining a similar canal-centering ability compared with continuous rotation [39].

This study has several limitations, despite its merits. Resin canals were used to control for anatomical variations and differences in dentin hardness, providing a more standardized analysis. However, owing to their softer and smoother texture, resin canals may require less force to cut than natural teeth [40], necessitating careful interpretation of our results. Additionally, although a wide range of NiTi instruments exist with varying thermal treatments, cross-sectional designs, and recommended applications, the present study focused solely on two NiTi instruments: PTG and PTU. However, this controlled approach minimized the impact of confounding geometric factors, thereby enabling a clearer assessment of the effects of downward load in thermally treated and non-treated instruments. The finding that instrumentation with PTG was less affected by downward load highlights thermal treatment as a key factor improving the instrument’s response to such forces. While this observation may have relevance for other thermally treated NiTi systems, further studies using extracted teeth or instruments with different mechanical properties are necessary to deepen our understanding of how downward load influences NiTi instrumentation.

The findings of this study highlight the importance of adapting the instrumentation technique based on the mechanical properties of NiTi instruments. Instruments with higher flexibility, such as PTG, offer a more forgiving and efficient approach, while those with lower flexibility, such as PTU, require careful consideration of downward loads and proper operator training. Further research is necessary to establish definitive guidelines for instrument selection and manipulation in clinical practice.

## 5. Conclusions

In the non-thermally treated PTU instruments., larger downward loads led to increased screw-in force, whereas smaller downward loads caused apical canal deviation and ledge formation. These effects were not observed in the thermally treated PTG instruments.

## Data Availability

The original contributions presented in this study are included in the article. Further inquiries can be directed to the corresponding author.
